# Combining EEG signals from the 2 members of a team to improve event identification^☆^

**DOI:** 10.1016/j.ynirp.2026.100356

**Published:** 2026-05-20

**Authors:** Jon M. Fincham, Shawn Betts, John R. Anderson

**Affiliations:** Department of Psychology, Carnegie Mellon, United States

**Keywords:** EEG, Collaborative tasks, Classification, Event detection

## Abstract

We examined the potential of combining EEG signals from multiple individuals to identify critical events in a team task. In this study two subjects played a video game in which they had complementary roles, one player serving as a Bait to distract 5 enemy fortress and the other serving as a Shooter to destroy the fortress. Twenty-one pairs of subjects were analyzed. Critical events, destruction of the fortress and deaths of each player, evoked distinguishable P300-like responses from both players. Fortress kills could be best identified by combining the two EEG signals, while deaths could be best identified by focusing on the response of the player who died. Hidden semi-Markov models (HSMMs) achieved good identification of the events by combining information about the temporal distribution of these critical events with the conditional probability of the EEG activity. These findings indicate that we can track and improve by adaptively merging or selecting the signals from different team members.

## Highlights

• Combining EEG signals improves detection of critical events in a team-based task.
• Hidden semi-Markov models enhance event localization.
• 85% of critical events are identified to within 50 msec.
• Different critical events produce distinguishable P300 responses.

## 1 Introduction

The goal of this research is to use EEG to identify critical events that happen when team members interact in a complex environment. Compared to other imaging methods, EEG offers the advantage of high temporal resolution and practicality. It has been widely employed in controlled laboratory settings to monitor processes occurring in trials lasting only a few seconds. In contrast, our focus is on monitoring cognition during longer, more open-ended tasks where events arise from interactions between multiple agents and their environment. In these scenarios, there is not a single correct sequence of actions or even a few options, but rather a vast array of possible action paths and environmental responses. These tasks allow us to understand the structure of complex cognition (Anderson et al., 2024, Cohen and D’Esposito, 2016, Dayan, 2008, Funke, 2010). Learning how to apply EEG in such contexts is valuable for developing brain–computer interfaces (for reviews, see Abiri et al., 2019, Lotte et al., 2018). Additionally, it can be used to tailor work environments to an individual’s current state (e.g. Yuksel et al., 2016, Dehais et al., 2019) and to monitor daily activities (Mihajlović et al., 2014, Baldwin and Penaranda, 2012).

While the more common use of EEG in open-ended tasks has been identifying relatively sustained states, such as fatigue during driving (e.g. Jap et al., 2009) or workload while managing a system (Baldwin and Penaranda, 2012), our focus is on pinpointing when specific cognitive events happen. Success at such temporal identification can be used to better align the actions and decisions of members of the team (which might well include artificial agents). We have had some success at tracking an individual player of a video game (Anderson et al., 2020).

The current study investigates the potential of combining the EEG signals from two individuals in a joint task. There has been substantial interest using simultaneous EEG (and other imaging modalities) to understand social cognition (Hasson et al., 2012, Czeszumski et al., 2020). While the focus in such research has been to identify the degree to which interacting individuals have synchronized EEG signals, our interest is in the degree to which we can achieve better identification of critical events, taking advantage of distinct information in signals that might not be synchronized. Each player has their own perspective on the state of the game, and more reliable event identification could be achieved by combining their EEG. It has been found that better identification is obtained using multiple subjects in single-trial brain–computer situations (Wang and Jung, 2011, Zheng et al., 2020)

Video games are increasingly popular tools for studying cognition (Altarelli et al., 2020, Bediou et al., 2023, Boot, 2015, Gray, 2017). Many video games exemplify open-ended tasks, where behavior emerges from the interaction between the game software and the players. These games often involve the learning of complex strategies and perceptual-motor skills. The video games we have examined involve a high rate of player action, providing a solid ground truth for evaluating the accuracy of EEG tracking.

There has been work on EEG and video games (Vasiljevic, 2020 for a review) but typically focused on using traditional BCI methods to serve as a controller for the game. Many of these applications leverage variants of the P300 (Verleger, 2020), which is sensitive to the occurrence of rare events. We have similarly used P300-like responses to track critical events in single-player video games (Anderson et al., 2020). The current paper will also focus on using P300-like responses, one from each team member, to identify critical events in a team game. We would like to improve identification of such events for potential application to improved team coordination.

**Fig. 1:**
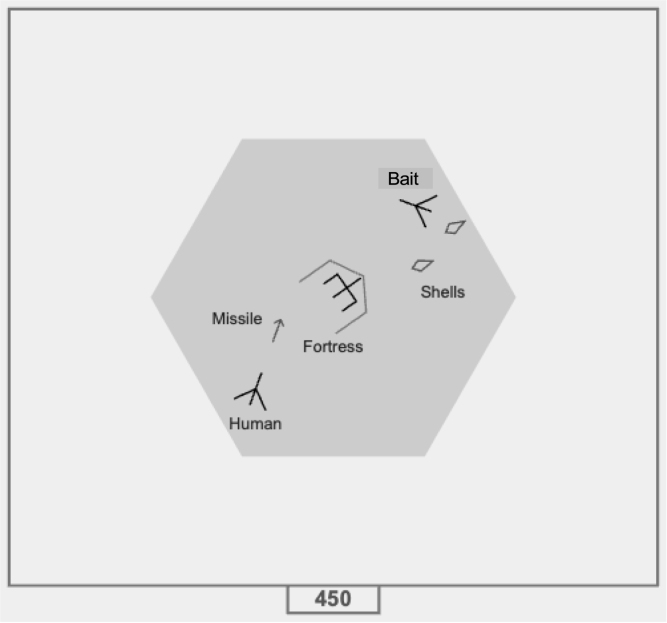
The Co-op Space Fortress screen, showing the hexagonal battle area with the Fortress in the middle. The Fortress has turned and has shot shells at the Bait. The Shooter is behind the Fortress and has shot a shell at the exposed rear of the fortress.

### 1.1 Co-op space fortress

Dimov et al. (2023) developed a 2-player game called Co-op Space Fortress to study the emergence of collaboration between two players. In Co-op Space Fortress, illustrated in Fig. 1, two players control spaceships which work together to destroy a fortress situated at the center of a grey hexagon.^1^ The key challenge in Co-op Space Fortress is that successful destruction of the fortress requires coordinated action between the two players. The fortress is protected by a shield that is impervious to missile attacks, except when the back portion of the shield temporarily disappears as the fortress fires at one of the ships. In this scenario, one player, known as the Bait, lures the fortress into lowering its shield by provoking it to fire. This exposes the back of the fortress, allowing the second player, the Shooter, to maneuver behind the fortress and launch a successful missile strike. The players are in different rooms and cannot communicate but they see the same game state which includes each other’s ship.

The spaceships operate in a frictionless environment and are controlled using four keys: W for thrust, D and A for clockwise and counterclockwise turns respectively, and the space bar to fire missiles. The frictionless environment makes navigation counterintuitive; for instance, the ship will not stop unless the player turns 180 degrees from the flight direction and thrusts for just the right amount of time. Learning to navigate under these conditions is a significant challenge, and it is further complicated in Co-op Space Fortress by the need for open and dynamic flight paths, which depend partly on the actions of the other player.

At the start of each game, the players begin outside the grey hexagon, positioned to the left and right of it. Both must enter the hexagon, as it defines the effective range for both fortress shells and ship missiles. The fortress will not target the Bait while it remains outside the hexagon, and the Shooter cannot destroy the fortress from outside the hexagon. Successfully destroying the fortress earns the players 100 points, after which both must exit the hexagon before the fortress respawns and they can attack again. Players lose 100 points if their ship is hit by the fortress, crashes into the fortress, or collides with the outer boundary of the game. After a ship is destroyed, it respawns one second later at the starting position. Additionally, players lose 10 points for every missile fired that does not result in the fortress’s destruction.

## 2 Method

### 2.1 Subjects and procedure

A total of 70 subjects were recruited from the university population of students and staff between the ages of 18 and 40. All were right-handed. None reported a history of neurological impairment. All subjects signed an informed consent. The experimental procedure and data handling was approved by the ethics committee of Carnegie Mellon University.

There were two sessions, the first involving 10 3-min games and the second involving 20 3-min games. Subjects practiced the role of Bait and Shooter for 10 games with an artificial partner in the first session, which served to familiarize them with the game and how to fly the plane in frictionless space. If they performed well enough, they were invited for a second session to be paired with a human partner to play 20 games with EEG data collection. To qualify for pairing a subject had to average at least 200 points in their last 3 games with the artificial agent. 36 subjects were recruited for the Shooter role and 30 passed the threshold. 34 subjects were recruited for the role of Bait and 31 passed the threshold. Fifteen of the 61 subjects who passed either chose not to go on or failed to show up for the experimental session leaving 23 pairs of subjects who were run. Two pairs were lost due to issues with the recording, leaving 21 pairs who serve as the basis for the analysis that will be reported.

The first 10 training games were played at the individual subject’s pace and lasted with instructions between 35.1 min and 49.1 min with a mean of 40.1. The subjects who played the second 20 games played at a pace chosen by the pair (the games began when both subjects indicated they were ready) and lasted between 65.3 min and 75.5 min with a mean of 68.7. Subjects were paid $10 for participation in the first behavioral session of 10 games. In addition, they earned a bonus for performance (4 cents per 100 points, total bonus range $0.16 to $7.49 with an average of $2.69). Subjects were paid $50 for participation in the second EEG session of 20 games. In addition, they earned a bonus for performance (4 cents per 100 points, total bonus range $2.66 to $17.79 with an average of $9.46).

### 2.2 EEG analysis and event identification

We had available to us two different EEG systems, one with wet electrodes and one with dry electrodes. While our decision to use two different systems was one of necessity, it does allow a comparison of results across systems, which will be discussed in Appendix. The two systems were:

1. EEG was recorded from 64 Ag-AgCl sintered electrodes (10–20 system) using a Biosemi Active II System (Biosemi, Amsterdam, Netherlands). The EEG was re-referenced online to the combined common mode sense (CMS) and driven right leg (DRL) circuit. Electrodes were also placed on the right and left mastoids. Scalp recordings were algebraically re-referenced offline to the average of the right and left mastoids. The EEG and EOG signals were digitized at 512 Hz and were subsequently filtered with a bandpass filter of 0.1 to 40.0 Hz. The vertical EOG was recorded as the potential between electrodes placed above and below the left eye, and the horizontal EOG was recorded as the potential between electrodes placed at the external canthi.
2. EEG was recorded from 21 electrodes (10–20 system) using a dry sensor system (DSI-24, Wearable Sensing, Inc., USA). Electrode positions according to the 10/20 international system consisted of 19 scalp locations (FP1, FP2, F7, F3, Fz, F4, F8, T3/T7, C3, Cz, C4, T4/T8, P7, P3, Pz, P4, P8, O1, and O2)^2^ and the left and right mastoids (A1, A2). The EEG signals were sampled at 300 Hz. Scalp recordings were algebraically re-referenced offline to the average of the left and right mastoids and were subsequently filtered with a bandpass filter using low- and high-cutoff frequencies of 0.1 Hz and 40.0 Hz, respectively.

11 of the 21 pairs had the Shooter assigned to the Biosemi system, and the Bait assigned to Wearable Sensing, while this was reversed for the remaining 10 pairs.

For both systems the EEG signal was recorded continuously for the entire experimental session and broken into 3-min games. To match the rate of game recording, the data were down-sampled to 60 Hz with default EEGLab (Delorme and Makeig, 2004) anti-aliasing filtering applied (FIR low-pass filter, 45 Hz cutoff frequency (−6 dB) and 10 Hz transition bandwidth. While the down-sampling loses high frequency information, we have found that this is not useful for event identification (Anderson et al., 2020). We have taken the path of doing minimal preprocessing on these data. While traditional steps of preprocessing (such as performing ICA and removing components correlated with oculomotor artifacts) can be important for accurately testing scientific hypothesis, we have found that it invariably removes signal and degrades classifier performance.

For each critical event, we extracted EEG data from 61 time points spanning a 1-s window centered on the event (−0.5 to ＋0.5 s), yielding a 61 by n electrode matrix. For each subject, these matrices were averaged separately for Kills, Shooter Deaths, and Bait Deaths, resulting in three condition-specific 61 by n matrices. Across the 21 teams, this produced 21 such triplets for Shooters and 21 for Baits. These averaged matrices formed the basis for visualization of electrode activity and subsequent statistical analyses.

To examine P300 responses, analyses focused on the 0.25–0.50 s post-event corresponding to 16 time points per electrode. Mean activity was computed for each subject as the average of the three central electrodes (FZ, CZ, and PZ over this period). We identified the time point of maximal positive amplitude in this interval for each of the three central electrodes. The peak latency was obtained by averaging these times across the three electrodes.

Both mean activity and peak latency were summarized for each combination of team (21), role (Shooter, Bait), and event type (three critical events). Separate within-team analyses of variance were conducted for mean activity and peak latency. Prior studies suggest a standard deviation of approximately 9 microvolts and a within-subject correlation of approximately 0.5. With 21 teams we would be well powered (80%) to detect differences on the order of 8 microvolts between conditions.

For purposes of classification, the EEG values of each electrode were z-scored for each game to standardize them across games. A one-second window around each game tick (30 game ticks before, the game tick, and 30 game ticks after) was used to identify when a game tick contained a critical event. This means that each game tick had associated with it a vector of 61*n electrode readings where n is the number of electrodes for that subject. These vectors represent regional effects, frequency effects (below 30 Hz), and their interactions. Because the vector associated with a game tick requires a signal for 1 s, game ticks at the beginning and end of a game do not have corresponding vectors. To reduce dimensionality and filter out noise, the vectors from all games were subject to a principal component analysis (PCA). We kept the top 50% of the PCA dimensions (1952 for Biosemi and 580 for Wearable Sensing). On average, the dimensions kept represented 99.0% of the variance for the 64 electrodes and 98.1% for the 19 electrodes.

We used the EEG vectors associated with ticks to identify which ticks involved one of the critical events: destruction of the fortress, death of the Shooter, and death of the Bait. Only 0.14% of the game ticks are critical events. Our general approach was to use training data from other games to estimate conditional probability densities that would be used to classify the game ticks in a held-out target game. These densities were estimated under two training regimes: (1) within-team identification, using data from other games played by the same team, and (2) between-team identification, using data from games played by different subjects performing the same role. We assumed that these densities had multidimensional normal distributions and used the training data to estimate separate means for the three critical events and for non-critical (Null) ticks. A single covariance matrix was estimated. For each tick in the target game, we could then compute the probabilities of observing its EEG vector conditional on it corresponding to one of the four types of events.

We were interested in the benefits of combining information from the two players. If we denote the conditional probability of the EEG vector on tick j given category i as $P\left(Shooter_{j}|i\right)$ for the Shooter and $P\left(Bait_{j}|i\right)$ for the Bait, the following is the formula for combining the two:

$$\frac{P\left(Shooter_{j}\left|i\right)\times P\left(Bait_{j}\right|i\right)}{\overset{n}{\underset{i}{\sum }}P\left(Shooter_{j}\left|i\right)\times P\left(Bait_{j}\right|i\right)}$$

where n is the number of categories.

As we will describe, these conditional probabilities can be used to classify ticks in various ways. For example, binary classification can be achieved by assigning ticks to one of two categories based on whether the ratio of their conditional probabilities exceeds a threshold. These probabilities also serve as inputs to hidden semi-Markov models for identifying critical events in the game.

**Fig. 2:**
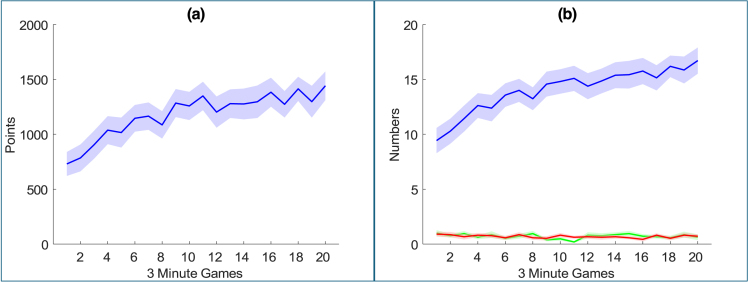
Mean values (lines) and standard errors (area around lines) per game for (a) points and (b) components that contribute to the point score.

## 3 Results

Fig. 2 shows various measures of performance during the 20 EEG games for the 21 subject pairs who met the selection criterion. Part (a) shows the growth in points per game. Points mainly reflect kills (＋100 points) and deaths (−100 points). Part (b) shows the mean number of kills and Shooter deaths and Bait deaths. The growth in kills is the main driver of the growth in points. Kills average 14.1 per game and show a highly significant increase over games (t’s(20) = 11.57, p < 0.0001^3^). On the other hand, both Shooter and Bait deaths average about 0.7 per game and have non-significant decreases over games (t(20) = −0.51 for Shooter deaths and −1.18 for Bait deaths).

**Fig. 3:**
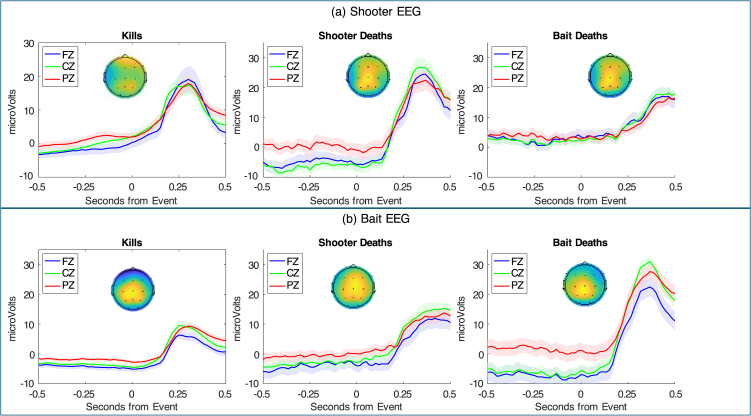
Response to 3 critical events for Shooter (a) and Bait (b): EEG activity (mean values and standard errors) of 3 central electrodes from a half second before a critical event to a half second afterwards. The scalp profiles show the distribution of activity averaged between 0.25 s to 0.5 s. They are scaled to range from zero to the maximum value.

Fig. 3 displays the EEG response from a half second before the event to a half second after the event, separately for Shooters (part a) and Baits (part b). These results are averaged over the wet and dry systems. As the Appendix discusses, the responses of the two systems are very similar, with the wet system giving a bit more reliable signal.

All the responses in Fig. 3 can be seen as P300s that vary in magnitude and time of their peaks. Table 1 gives the mean activity of the 3 central electrodes 0.25 to 0.50 s after the event and the points of peak activity (calculated for each central electrode and then averaged). We analyzed the reliability of the differences in Table 1 over teams (pairs of subjects):

**Table 1:** P300 responses to critical events by Shooters and Baits.

• **Mean Activity**. There is a highly significant role-by-event interaction (F(2, 40) = 27.99, p < 0.0001). Shooters are more positive than Baits after Kills (t(20) = 3.78, p = 0.001), and after Shooter Deaths (t(20) = 2.82, p = 0.011), but less positive after Bait Deaths (t(20) = 3.59, p = 0.002). The death comparisons correspond to the assumption that deaths are more important to that player. One might argue that Kills are more important to the Shooter whose actions are more directly responsible. Shooter’s response to their own deaths is greater than their response to either a Kill (t(20) = 2.99, p = 0.007) or to a Bait Death (t(20) = 3.65, p = 0.002). The magnitude of Shooter responses does not differ significantly between a Kill and a Bait Death (t(20) = 0.30). Baits show greater response to their own deaths than either a Kill (t(20) = 8.04, p < 0.0001) or to a Shooter Death (t(20) = 4.88, p < 0.0001). Their response to a Shooter Death is greater than their response to a Kill (t(20) = 3.57, p = 0.002).
• **Peak Activity.** In a dynamic environment like this game where multiple things are changing, the point of peak activity may reflect average differences in when the critical event is noticed by the player. Peak activity also shows a highly significant role-by-event interaction (F(2, 40) = 19.55, p < 0.0001). Shooters respond sooner than Baits after Kills (t(20) = 2.47, p = 0.023) and after Shooter Deaths (t(20) = 2.99, p = 0.007), but slower after Bait Deaths (t(20) = 5.12, p < 0.0001). Both players respond sooner to Kills than either Shooter Deaths (t(20) = 5.69, p < 0.0001 for Shooters; t(20) = 6.75, p < 0.0001 for Baits) or Bait Deaths (t(20) = 5.12, p < 0.0001 for Shooters; t(20) = 2.42, p = 0.025 for Baits). Players respond sooner to their own death than that of the other player (t(20) = 4.17, p < 0.001 for Shooters; t(20) = 4.07, p < 0.001 for Baits).

### 3.1 Identifying critical events

The events driving the patterns in Fig. 3 are rare — only 0.131% of game ticks involve kills, 0.006% involve Shooter Deaths, and 0.006% involve Bait Deaths. To identify these critical events, we need to discriminate them from the many more Null ticks and from each other. Table 2 reports how well we can discriminate among the four kinds of ticks: Null ticks, Kill ticks, Shooter Death ticks, and Bait Death ticks. In part (a) we use conditional probabilities estimated from other games of a team to make the discrimination. In part (b) we use conditional probabilities estimated from all other teams to make the discrimination. We report separately success using the Shooter’s EEG, success using the Bait’s EEG, and success using their combined EEG.

To classify a tick into one of two categories, we can compute the ratio of their conditional probabilities and assign the tick to a category if the ratio exceeds a selected threshold. Adjusting the threshold to favor one category increases the hit rate for that category but also raises the false alarm rate for the other. Table 2 presents a threshold-independent measure of discriminability based on the area under the Receiver Operating Characteristic (ROC) curve, which plots the true positive rate against the false positive rate across thresholds. AUC ranges from 1.0 for perfect discrimination, to 0.5 for chance-level performance, and below 0.5 when performance is worse than chance. Each sub-table in Table 2 reports the mean AUCs for the 6 possible pairwise discriminations between the 4 categories. Table 2 also gives the number of teams for whom the AUC is greater than 0.5, and the mapping of these counts onto significance levels according to a sign test.

**Table 2:** Area under the curve measures of miscriminability between different types of game ticks (in parentheses: number of pairs for whom the AUC is greater than 0.5)

While the mean AUCs are all above 0.5, there are substantial differences among them. As one might surmise from Fig. 3 and Table 1, the Shooter EEG is better than the Bait EEG for identifying Shooter Deaths versus Null ticks for either within-team training (16 of 21 teams) or between-team training (18 of 21 teams). Likewise, the Bait EEG is better than the Shooter EEG for identifying Bait Deaths for either within-team training (20 of 21 teams) or and between-team training (12 of 21 teams). The combined EEG is never significantly worse than the individual EEG at discriminations, always significantly better than the poorer individual EEG, and in some cases better than either single source. In particular, combined is better for discriminating Kills from Null ticks for within-team training (19 of 21 teams compared to Shooter EEG; 21 of 21 compared to Bait EEG).

With the exception of discriminating Kills from Null ticks, using between-team training is superior to using within-team training. Looking at the combined EEG, the number of 21 teams showing better results for between-team training is 17 for Shooter-Death versus Null, 13 for Bait-Death versus Null, 15 for Kill versus Shooter Death, 15 for Kill versus Bait Death, and 16 for Shooter Death versus Bait Death. The notable exception is discriminating Kills from Null ticks where within-team training is better for all 21 teams. The average AUC in this case, 0.994, is very close to 1. Given the relatively large number of Kills and the very large number of Null Ticks, it is possible to accurately estimate the team-specific nuances that better identify Kill ticks. In contrast, there are too few deaths per team to allow good within-team discrimination for comparisons that involve them.

**Table 3:** Mean number of ticks in a game classified as Kills versus Null ticks for true Null ticks and Kill ticks. Parts (a) and (b) give classification at 2 thresholds for labeling a tick as a Kill.

### 3.2 Identifying kills

While an AUC of 0.994 for distinguishing Kill versus Null ticks is very high, there are still many Null ticks mislabeled as Kills. Table 3a shows classification performance when a tick is labeled as a Kill whenever its conditional probability for Kill exceeds that for Null (i.e. when the ratio of conditional probabilities is greater than 1). Under this criterion, only 0.6% of Null ticks are misclassified, and 91.2% of Kill ticks are correctly identified (row-wise comparisons). However, due to the overwhelming number of Null ticks, 83.2% of all Kill labels are applied to Null ticks (second column). Raising the threshold to the point where posterior probabilities for Kill and Null are equal reduces false positives by half, as shown in Table 3b. Yet, even at this setting, most Kill labels still fall on Null ticks, and the proportion of correctly labeled Kill ticks drops to 76.6% . There is no threshold at which Kill ticks receive more correct labels than Null ticks receive incorrect ones.

Table 3 displays classifications based on the combined probability. Using the threshold of probability ratio greater than 0.5, Table 4 shows how the classification based on the Bait probabilities corresponds to classification based on the Shooter probabilities. Both in the case of Null ticks and the case of Kill ticks, the two EEG sources tend to agree on the correct classification The interesting observation is that when one source makes a mistake the other source tends to disagree. In the cases of disagreement, the majority of the classifications based on the combined data are correct: 92.93% of the disagreements on Null ticks and 81.3% of the disagreements on Kill ticks.

**Table 4:** Success at identifying critical events versus Null ticks. Kills are identified over the entire game using within-team training. Deaths are identified in segments between Kills using between-team training.

**Fig. 4:**
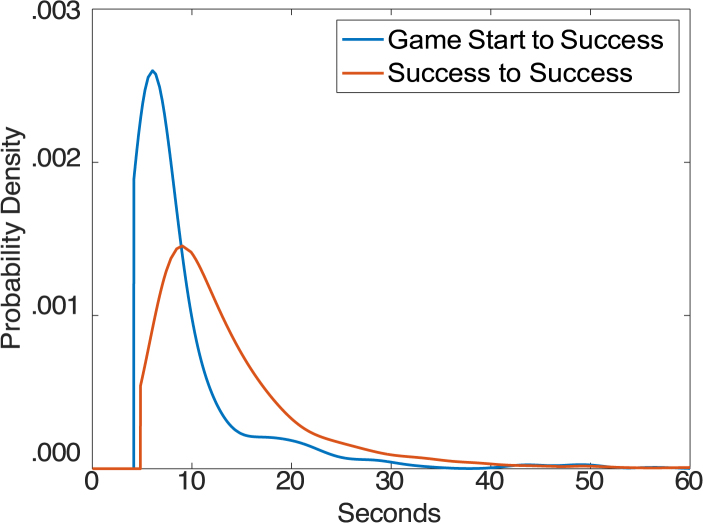
Distributions of times to Kills.

The challenge is to determine which of the ticks that look like Kills are actual Kills. We use a hidden semi-Markov model (HSMM, Rabiner, 1989) to serve this purpose. The HSMM combines the probabilities that various ticks are kills with information about when kills are likely to occur. Fig. 4 shows fitted distributions^4^ to the durations between a start and a Kill and between a Kill and a Kill. The mean duration between the start of the game and a Kill (9.9 s) is shorter than between 2 Kills (12.3 s). To go from the start of a game to a Kill both ships just need to fly in and get into position, while to go from Kill to Kill the ships must first fly out so that the fortress can respawn. These distributions tell the HSMM when Kills are likely and so can be used to reject many Kill placements that might look good from just the EEG. While the distributional information shown in Fig. 4 is estimated from all teams, the HSMM only used distributions estimated from other games of the pair in fitting the data for any game from the team. The HSMM Viterbi algorithm combines this distributional information with the conditional probabilities to identify the maximum likelihood placement of events in the target game. The Appendix specifies the likelihood being maximized.

We have developed a general measure of how well the HSMM was able to identify the placement of the Kills in a game as a combination of recall and precision measures (Buckland and Gey, 1994). The recall measure was the average temporal tick distance between true Kills and the closest predicted Kill. The precision measure was the average temporal tick distance between predicted Kills and the closest actual Kill. The mismatch measure we report is the average of these two measures. The distances going into these measures were bounded by 100 ticks. So, this measure could vary from 0 (all Kills and predicted Kills exactly matching) to 100 (worst possible mismatch). Excluding the one game where there was no Kill, the mean mismatch measure was 12.8 ticks, which equates to .21 s. The HSMM uses the probabilities estimated from the joint EEG signals. For comparison, the score if we used just the Shooter’s EEG is 19.6 (t(20) = 3.38, p = 0.003 for difference from using both EEG sources) and the score using just the Bait’s EEG is 41.2 (t(20) = 6.16, p < 0.0001).

**Fig. 5:**
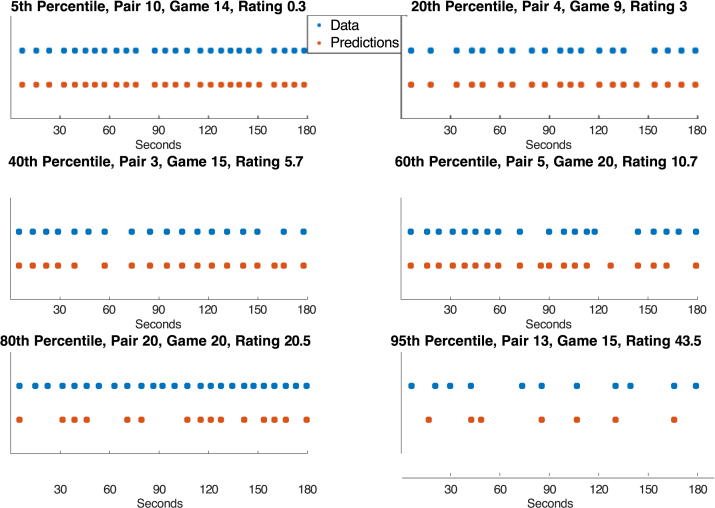
Examples of differential success at determining when kills occurred.

We calculated the mismatch score between each of the 419 games with Kills and the 419 reconstructions based on the combined EEG. The reconstruction for a game matched the actual game better than any other reconstruction for 413 of the 419 games. The mean rank of the reconstruction for a game among all games was 1.2 (chance would be 210). Fig. 5 illustrates the reconstruction of games whose ratings span from the 5th percentile (better scores) to the 95th percentile (worse scores). Even at the 95th percentile, some of the Kills are being closely predicted. Over all games 87.3% of all Kills are being identified to within 2 ticks.

### 3.3 Identifying deaths

We investigated how well we could identify deaths between the game segments defined by kills: the interval from the start of the game to the first kill, the intervals between kills, and the interval from the last kill to the end of the game. Of the 6332 such segments across teams there were 239 cases where a single Shooter death occurred in such a segment and 214 cases where a single Bait death occurred. We chose to focus on these cases. The segments involving a Shooter death averaged 25.5 s and the segments involving a Bait death averaged 24.2 s, much longer than the average segment (see Fig. 4). The challenge is to identify where the death occurred in these long intervals. We calculated the conditional probabilities of a death occurring on any particular tick using between-team training. Since conditional probabilities are based on the EEG in a 1-s interval surrounding the tick, we did not calculate conditional probabilities for ticks in the first half second or last half second of a segment. This effectively eliminates any confusion with EEG patterns in the immediate vicinity of a kill.

Table 5a shows AUC measures for discriminating Shooter Deaths from Null ticks and for discriminating Bait deaths from Null ticks. For comparison the Table also shows the measures for Kills using within-pair training. The AUCs shown here for deaths are somewhat better than the AUCs shown for distinguishing deaths from Null ticks in Table 3b, reflecting the elimination of Null ticks within 0.5 s of a Kill. However, they are still less than the very high AUCs for identifying Kills versus Null ticks using the combined EEG. Again, the Shooter activity is better than the Bait activity for identifying Shooter deaths (18 of 21 teams show greater AUCs) while Bait activity is better than Shooter activity for identifying Bait deaths (19 of 21 teams). There is a marginal tendency for the Combined activity of both to do better than just the Shooter for Shooter deaths and to do better than just the Bait for Bait deaths (in both cases 15 of 21 teams are in this direction, p = 0.074).

**Table 5:** Success at identifying critical events versus Null ticks. Kills are identified over the entire game using within-team training. Deaths are identified in segments between Kills using between-team training.

**Fig. 6:**
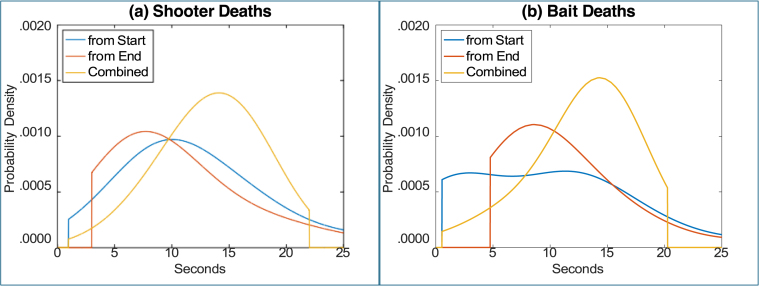
Distributions of times of deaths from the start of a Kill interval and from the end of a Kill interval. The combined distribution gives the probability of a death given that the Kill interval is 25 s.

We created a simple HSMM that combined the conditional probabilities with two temporal distributions, one from start of segment to Death and one from Death to end of segment. Fig. 6 illustrates these distributions. These two can be combined to determine the probability that a Death will occur anywhere in the segment (the prior that will be multiplied by the conditional probability of a death on that tick given the EEG). Fig. 6 also illustrates this combined distribution if the segment is exactly 15 s. Note the Start and End distributions are zero until some minimal duration. For Start distributions this reflects the minimal time after a kill until a death. For End distributions this reflects the minimal time from a death to the next kill. The Combined distribution rises from 0 at the minimal Start time and falls back to 0 at a point that is the minimal End time from 15 s In general, these distributions favor deaths in the middle of the segment. The one exception is the Start Distributions for Bait Deaths where there are many deaths just after a kill. These reflect cases where the Fortress fires a shell shortly before being destroyed and that shell hits the Bait shortly after the Fortress is destroyed.^5^

From these distributions and the death probabilities, the HSMM assigns the death to the most probable tick in the segment. Table 3b reports the percentage of cases that the predicted death is within 2 ticks of the actual death. By combining EEG data from both players, we achieve 57.7% accuracy in identifying Shooter Death to within 2 ticks and 63.6% accuracy for Bait Deaths. Using the same scoring scheme as for the Kill ticks, Table 3c shows the mean mismatch score for distance from the target tick. The combined EEG achieves mismatch scores of 25.8 for Shooter and 20.7 for Bait. These measures are not as good as the identification of Kills where 87.3% of the Kills are identified to within 2 ticks and mean mismatch score is 12.8 ticks. Still, it is much better than the best we could do without EEG. If we were to place the deaths at the point of the maximum combined distributions (Fig. 6) only 2.1% of the Shooter deaths would be placed within 2 ticks and only 2.3% of the Bait deaths. The mismatch scores would be 76.1 for Shooter deaths and 81.3 for Bait deaths.

### 3.4 Conclusions

Both members of a team showed distinguishable variants of P300s to the three critical events in the game (Kills, Shooter Deaths, and Bait Deaths). The players’ response to their own deaths was stronger than their response to kills or deaths of the other player. The response to deaths of either player was delayed relative to their response to kills. Combining the information from both players improves the identification of these critical events.

Given the rarity of deaths, identification of deaths was better given training on other teams than training of the other games of the pair. There were too few deaths per team to give as good within-team discrimination. On the other hand, given the fairly large number of Kills (an average of 281 per pair), using within-team training was better for discriminating them from Null ticks. An HSMM, using probabilities from within-team training on the combined EEG, resulted in the high placement of Kills illustrated in Fig. 5.

While much better than chance, an HSMM did not do as well in identifying deaths between Kill boundaries. Had there been more deaths we might have been able to take advantage of the benefits of within-team training for deaths. There is a strong negative rank-order correlation (r = −0.858, p < 0.0001) between the number of Shooter Deaths observed by a pair and the AUC advantage of between-team training over within-team training. The correlation is weaker but still significant (r = −0.511, p = 0.018) between the number of Bait Deaths and the between-team advantage for distinguishing Bait Deaths from Null ticks.

## 4 Discussion

Both members of a team showed distinguishable variants of P300s to the three critical events in the game (Kills, Shooter Deaths, and Bait Deaths). The HSMMs used this information and temporal information to identify when these events happened in the game. Traditionally, the P300 has been interpreted as reflecting context updating and the allocation of attentional resources to task-relevant events (Donchin and Coles, 1988), with its latency indexing stimulus evaluation time (Kutas et al., 1977). More recent work has reframed the P300 as a decision-related signal reflecting evidence accumulation and belief updating (Mars et al., 2008, O’connell et al., 2012). Generalizing from discrete trial paradigms to continuous interactive tasks, P300-like responses can be understood as indexing internally defined, goal-relevant event boundaries rather than stimulus onsets per se (Makeig et al., 2009, Polich, 2007)

While this research has been focused on mining EEG data to identify events, there is no reason to use it in isolation. We could expect better performance when combined with other temporally sensitive measures from team members such as eye movements (e.g. Coutray et al., 2025, Liu et al., 2025, Tan et al., 2021). There has been considerable work on the challenges and potential of combining eye movements with EEG (Schotter et al., 2025). Hidden semi-Markov models are particularly well suited to perform synthesis of multiple modalities. For instance, eye features like gaze position can be treated as time-varying features just as electrode activity. This would combine attentional information from the eyes with importance information from the EEG.

These results point to the advantage of combining EEG from team members in monitoring progress on a task. Individual team members will respond to what they regard as significant events with strong responses. Sometimes, one of the members will give a much clearer response to the event. The combined EEG takes advantage of the sensitivity of each participant in identifying that a significant event has happened. HSSMs, given the information from the combined EEG, did better than HSSMs given information from just one source. While our results are from a particular game, the advantage of combining EEG from multiple participants should apply to any team task.

In the current game both members of a team see the same game, but critical events in that game evoke somewhat different responses. In larger or more complex teams, such as in search and rescue, team members typically operate under partial observability. That is, different individuals have access to different pieces of information, and no single person sees the full picture. In such settings, team performance critically depends on integrating these disparate viewpoints into a shared situational awareness. Our work shows that improved event identification can be achieved by either combining signals from team members who have access to the event (as was the case for kills) or by using the response of the individual who gives the most reliable signals (as was almost the case for deaths).

This research has potential applications to improving team performance in time-critical, high-stakes environments such as aviation, military operations, emergency response, and complex medical procedures. By showing that critical task events are more reliably detected when EEG signals are combined, the work suggests a shift from individual-based monitoring to team-level monitoring. In practice, this could support adaptive systems that detect when a team has successfully registered an important event (e.g., a mission success or failure) or when a critical event has been missed by one or more members. Such systems could provide targeted alerts, redistribute workload, or adjust levels of automation based on the collective evidence.

Beyond its practical relevance, this research contributes to a theoretical understanding of cognition in collaborative settings. As argued by Anderson et al. (2024), the application of hidden semi-Markov models (HSMMs) to EEG data provides a powerful approach for tracking cognition in open-ended tasks where the timing and sequence of internal processes are not externally defined. By modeling cognitive states as latent variables with variable durations, this framework enables direct tests of competing cognitive theories that posit different processing sequences or strategies, even when observable behavior is similar. Different theories of teamwork predict distinct patterns of shared versus role-specific processing. By reconstructing cognitive trajectories of interacting individuals, this methodology provides a principled basis for selecting among competing models.

**Fig. B.7:**
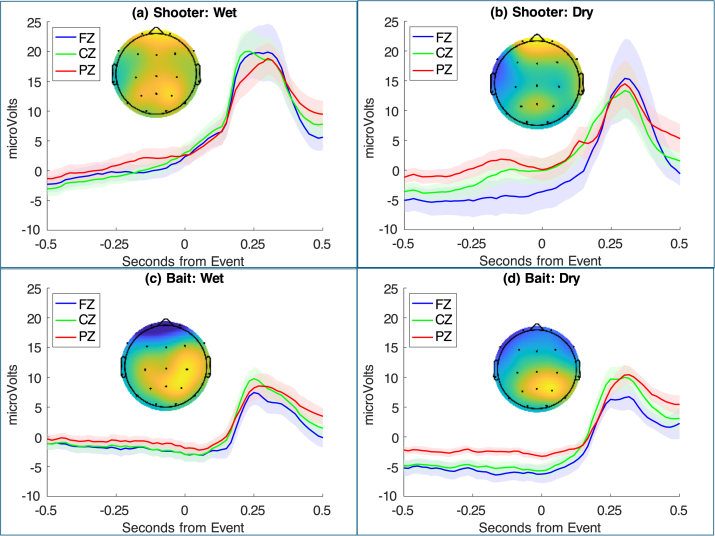
EEG activity (mean values and standard errors) of 3 central electrodes from a half second before a Kill to a half second afterwards. The scalp profiles are of the difference between average activity between 0.25 s to 0.5 s. They are scaled to range from zero to the maximum value.

## CRediT authorship contribution statement

**Jon M. Fincham:** Software, Methodology, Data curation. **Shawn Betts:** Writing – review & editing, Supervision, Software, Project administration, Methodology, Data curation. **John R. Anderson:** Writing – review & editing, Writing – original draft, Supervision, Software, Resources, Project administration, Methodology, Investigation, Funding acquisition, Formal analysis, Conceptualization.

## Declaration of competing interest

I have no completing interests, financial or otherwise.

## Figures and Tables

**Table B.6:** Comparison of wet and dry systems for Kills.

## Data Availability

Data is available at https://osf.io/efbux/.

## Appendix A Viterbi algorithm

In the case of Kills, the Viterbi algorithm finds the set of n game ticks $t_{i}$ that maximize the quantity:

$$D_{SK}\left(t_{1}\right)\times R\left(EEG\left(t_{1}\right)\right)\times \left(\overset{n}{\underset{i=2}{\prod }}D_{KK}\left(t_{i}-t_{i-1}\right)*R\left(EEG\left(t_{i}\right)\right)\right)\times S_{SK}\left(10800-t_{n}\right)$$

where

• $D_{SK}\left(t\right)$ is the probability of t ticks from Start to first Kill.
• $D_{KK}\left(t\right)$ is the probability of t ticks from Kill to Kill.
• $S_{KK}\left(t\right)$ is the probability of more than t ticks between two Kills.
• R(EEG(t)) is the ratio of two conditional probabilities: (1) the probability of the EEG signal on tick t if a Kill occurred on that tick, (2) the probability of the EEG signal on tick t if a Kill did not happen. As discussed in Anderson et al. (2020), using ratios allows us to ignore the probability of the signal between critical events. It also deals with the non-independence of adjacent ticks.

## Appendix B Comparison of wet and dry EEG systems

Given that kills were much more abundant that deaths, we compared the response of the wet and dry systems at the point of kills. Fig. B.7 shows the activity obtained from the 4 types of subjects defined by the cross of the electrode system and the role of the player. We calculated the mean activity of the 19 electrodes common to both systems in the interval 0.25 to 0.5 s after a Kill. Table B.6a shows the correlation of these patterns between different types of subjects. Wet Shooters have similar patterns of activity to Dry Shooters and similarly for Wet and Dry Baits. On the other hand, the patterns are not correlated across roles. Shooter activity is maximal for frontal electrodes while it is maximal at central and posterior electrodes for Baits.

Comparable to the two parts of Table 1, Table B.6b gives mean activity over all electrodes in the period 0.25 to 0.5 s after a kill and Table B.6c gives the mean time of peak activity. Both factors show a significant difference between the two roles (F(1,38) = 5.18, p = 0.029 for mean activity; F(1,38) = 7.60, p = 0.009 for peak activity), but not a significant difference between the two systems (F(1,38) = 0.84, p = 0.366 for mean activity; F(1,38) = 0.85, p = 0.362 for peak activity). The interactions of these two factors are also not significant. (F(1,38) = 1.64, p = 0.209 for mean activity; F(1,38) = 0.31, p = 0.578 for peak activity).

Table B.6d presents the AUC measures of discriminability for the 4 types of subjects. Shooters are more discriminative than Baits (F(1,38) = 20.09, p < 0.0001) and the wet system is more discriminative than the dry (F(1,38) = 9.68, p = 0.004). The interaction is not significant (F(1,38) = 0.59, p = 0.448). With respect to the combined activation, pairing a dry Shooter with a wet Bait is marginally better than a wet Shooter with a dry Bait (AUCs of 0.997 vs 0.990; t(19) = 1.78, p = 0.091).

## Footnotes

2 These are among the scalp electrodes for the Biosemi system.

## Footnotes

1 Demo available at https://osf.io/efbux/.

3 These are tests of linear slope. All t-tests reported are 2-tailed.

4 Using the Matlab function ksdensity.

5 Shells persist 3 s after being fired.
